# Infodemiological Examination of Personal and Commercial Tweets About Cannabidiol: Term and Sentiment Analysis

**DOI:** 10.2196/27307

**Published:** 2021-12-20

**Authors:** Jason Turner, Mehmed Kantardzic, Rachel Vickers-Smith

**Affiliations:** 1 Data Mining Lab Department of Computer Science and Engineering University of Louisville Louisville, KY United States; 2 Department of Epidemiology College of Public Health University of Kentucky Lexington, KY United States

**Keywords:** social media, social networks, text mining, CBD, cannabidiol, cannabis, public health, drug regulation, Twitter, sentiment analysis, unregulated substances

## Abstract

**Background:**

In the absence of official clinical trial information, data from social networks can be used by public health and medical researchers to assess public claims about loosely regulated substances such as cannabidiol (CBD). For example, this can be achieved by comparing the medical conditions targeted by those selling CBD against the medical conditions patients commonly treat with CBD.

**Objective:**

The objective of this study was to provide a framework for public health and medical researchers to use for identifying and analyzing the consumption and marketing of unregulated substances. Specifically, we examined CBD, which is a substance that is often presented to the public as medication despite complete evidence of efficacy and safety.

**Methods:**

We collected 567,850 tweets by searching Twitter with the Tweepy Python package using the terms “CBD” and “cannabidiol.” We trained two binary text classifiers to create two corpora of 167,755 personal use and 143,322 commercial/sales tweets. Using medical, standard, and slang dictionaries, we identified and compared the most frequently occurring medical conditions, symptoms, side effects, body parts, and other substances referenced in both corpora. In addition, to assess popular claims about the efficacy of CBD as a medical treatment circulating on Twitter, we performed sentiment analysis via the VADER (Valence Aware Dictionary for Sentiment Reasoning) model on the personal CBD tweets.

**Results:**

We found references to medically relevant terms that were unique to either personal or commercial CBD tweet classes, as well as medically relevant terms that were common to both classes. When we calculated the average sentiment scores for both personal and commercial CBD tweets referencing at least one of 17 medical conditions/symptoms terms, an overall positive sentiment was observed in both personal and commercial CBD tweets. We observed instances of negative sentiment conveyed in personal CBD tweets referencing autism, whereas CBD was also marketed multiple times as a treatment for autism within commercial tweets.

**Conclusions:**

Our proposed framework provides a tool for public health and medical researchers to analyze the consumption and marketing of unregulated substances on social networks. Our analysis showed that most users of CBD are satisfied with it in regard to the condition that it is being advertised for, with the exception of autism.

## Introduction

Although the cannabis plant has been used as a medication for centuries, the use of cannabis was criminalized in the United States in 1937. However, beginning in the 1990s, a few states began allowing the medical use of cannabis even as the plant remained illegal at the federal level [1]. As more states introduced laxer cannabis policies, public interest in the medicinal properties of cannabis evolved to embrace cannabidiol (CBD). CBD is an active chemical found in variants of the cannabis plant that does not have the psychoactive side effects of the tetrahydrocannabinol (THC) components of the cannabis plant [2].

In recent years, the utility of medical cannabis has grown as a major point of discussion in public policy, and especially in online social media discourse [3,4]. In particular, consumers have reported using CBD to treat various conditions, including epilepsy and other neurological disorders, insomnia, and some mental illnesses. CBD remains unregulated by the Food and Drug Administration (FDA) and has not been subjected to the same conclusive trials as most medications pertaining to its many specific uses. In fact, the FDA has only approved one cannabis-derived medication and three cannabis-related drugs to date—all of which require a prescription—and it has not approved marketing cannabis as a safe and effective drug for treating any disease [5]. Palmieri et al [6] reported promising results of CBD as a treatment for inflamed skin conditions and scars. There have also been many studies performed on CBD as a treatment for anxiety and sleep disorders [7-10], as a pain reliever [11-13], and as a treatment for cancer and cancer side effects [13-15].

Although most CBD-based medications and nutritional supplements have not demonstrated safety and efficacy for the numerous indications for which they are used, there are nevertheless many claims in public circulation regarding the effectiveness of CBD for a wide spectrum of ailments.

Social media serves as a primary location for viral CBD marketing and individuals sharing their experiences of personal use. Twitter, in particular, is a useful platform for understanding how cannabis, including CBD, is marketed to consumers and how individuals are using cannabis, as it provides a large corpus of both personal and commercial tweets [3]. Additionally, sentiment analysis can be used on personal and commercial CBD tweets to gauge user satisfaction for CBD treatment of particular medical conditions.

Thus, we propose a framework for the use of text mining in social networks that can help public health experts understand the landscape of personal and commercial claims and sentiments about unregulated substances such as CBD. There are two practical advantages to this framework. First, the data are readily available, easy to access, and inexpensive to use compared to administering surveys or utilizing data from governments and health providers. Second, public health researchers have already shown that sentiment analysis is an effective tool for understanding the public perception of drugs, diseases, and medical services as well as for detecting certain forms of depression [16-19].

To demonstrate the usefulness of this framework, we distinguished between tweets reflecting personal CBD use and tweets reflecting the sales, promotion, and commercialization of CBD. The two resulting corpora of tweets were analyzed for medical-related terms such as conditions, side effects, anatomical terminology, and use of other substances mentioned. This approach allowed us to identify terms that are being used in online CBD marketing in relation to the terms referenced by individuals taking CBD. In addition, we performed sentiment analysis on personal CBD tweets to gauge public opinion on the effectiveness of CBD in treating certain conditions. We were also able to use the Valence Aware Dictionary for Sentiment Reasoning (VADER) model to compute the sentiment of the personal and commercial CBD tweets making reference to specific medical conditions. The VADER sentiment model was used as it was specifically developed for analyzing sentiment on posts made on social network sites such as Twitter [20]. Since its development, VADER has been used to gauge sentiment on digital assistants among Twitter users, political tweets, as well as course evaluations [21-24]

Findings from this study will provide important information on public perceptions of CBD and how CBD is marketed through a social media platform. Misinformation has increased with the rise of social media. Recently, researchers have used social networks to analyze these concepts with regard to medical misinformation [25-28], which can contribute to negative health outcomes. For this reason, unverified claims overstating, exaggerating, and lying about CBD’s medical benefits have circulated freely on the internet, leading many to wonder how to assess the actual benefits (if any) of using CBD in medical contexts. Some of the conditions referenced in these claims include ear pain in infants, autism, attention deficit and hyperactivity disorder, Parkinson disease, and Alzheimer disease [29]. By analyzing personal and commercial tweets about CBD using the adaptable and generalizable framework developed for this study, public health and medical professionals can more readily identify viral misinformation pertaining to CBD claims.

## Methods

### Framework Development

**Figure 1:**
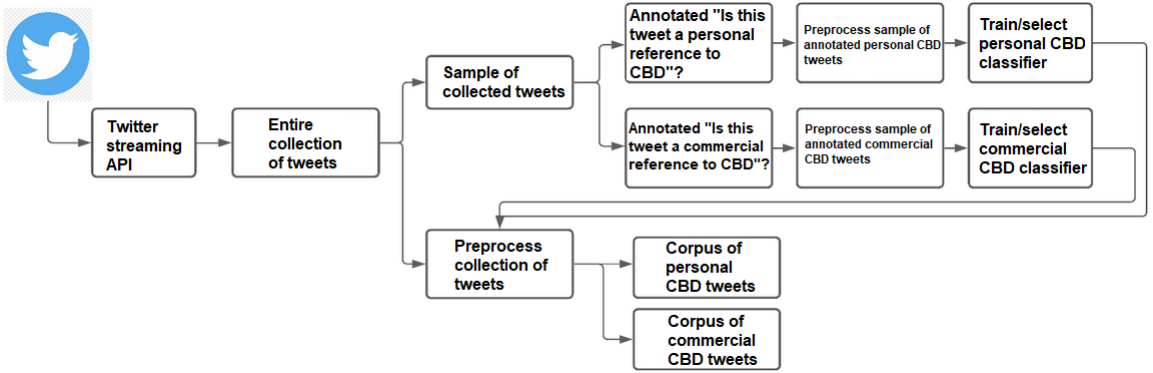
Workflow of Tweet collection and classification. API: application programming interface; CBD: cannabidiol.

To build this framework, we collected tweets that make reference to “CBD” or “cannabidiol.” We then took a random sample of these tweets and labeled them as a personal CBD reference (true/false) or commercial CBD reference (true/false). Using these annotated tweets, we trained two binary text classifiers to separate the personal and commercial CBD tweets from the larger collection of tweets. Using these two corpora of tweets, we identified the most frequently occurring medically relevant terms (terms related to diseases, conditions, symptoms, body parts, other substances, cannabis, etc) and compared these term frequencies between the two corpora. Figure 1 provides a visual description of our framework from data collection through classification. We then computed the sentiment of the personal CBD tweets containing specific terms using the VADER model to assess the satisfaction of CBD for treating those conditions.

### Data Collection

We collected tweets from the Twitter public stream using the Tweepy Python package as an interface to the live Twitter stream, which provides access to approximately 1% of the public tweets as they are created. Our data collection ran from October 7, 2019, to January 26, 2020, and used the search terms “CBD” and “cannabidiol.” We selected this period as it represents the time when CBD had become popular. We restricted our collection to an approximate 3.5-month period so that we could collect a sufficient amount of tweets within a window of time to avoid potential concept drift within the data. We also set filters to collect tweets that were written in English and were original (ie, no retweets). We did not want to include retweets as the actual contents of these tweets are linked to another author and are nearly identical in text to an existing tweet. For each tweet collected, we kept the full-length tweet text, the ID of the tweet, the time the tweet was created, and the Twitter user that authored the tweet. The resulting collection consisted of a data set of 567,850 tweets.

### Tweet Annotation

To identify the personal and commercial-related CBD tweets from our collection of 567,850 tweets, we built two binary classifiers trained on a sample of 5496 tweets. This sample of tweets was obtained by taking a 6000-tweet sample from our collection and removing entries with verbatim duplicate tweets. The process of annotating the personal CBD tweets consisted of evaluating each tweet in the sample as to whether or not it was posted from an individual (ie, not a “bot”) discussing the past, current, and/or future use of CBD. The process of annotating the commercial CBD tweets consisted of evaluating each tweet in the sample as to whether or not it was posted from an actual (ie, not a “bot”) nonnews entity selling, advertising, or promoting CBD. These classifiers were used to distinguish between personal and commercial CBD-related tweets. To train these classifiers, all tweets in the sample were manually labeled as either a personal CBD-related tweet or a nonpersonal CBD-related tweet, and either a commercial CBD-related tweet or a noncommercial CBD-related tweet, according to the content in their full text, which consisted of a maximum of 280 characters. Textbox 1 provides some examples of the personal and commercial CBD-related tweets, and Textbox 2 provides some examples of the types of tweets referencing CBD that we encountered that were considered erroneous for both the personal and commercial CBD classes.

**Examples of personal and commercial cannabidiol (CBD)-related tweets (paraphrased slightly for anonymity). CBC: cannabichromene.**

**Personal CBD-related tweets.**

CBD products are so good for anxiety, and they don’t make you high

I’ve used CBD for anxiety. It is WAY healthier than taking benzodiazepines…I also use CBC for pain. You know what else is bad for your liver? Tylenol and Ibuprofen

Take some painkillers with sleeping aid like Tylenol or Advil PM or something…any CBD or weed maybe try that too

CBD gummies will not give you the high, but for me personally CBD oil edibles helped with anxiety and menstrual cramps

**Commercial CBD-related tweets**

Go Away!!!!Pain! We have a variety of CBD products for your needs.... Make sure to ask about our selection your next visit. URL

Over time, poor sleep can leave you feeling wrecked...Could CBD help? URL #cbd #cbdoil #hemp #cannabis #sleep #insomnia

Chronic Fatigue...Cannabis CBD THC oil – URL

Our CBD cream combines the relief potential of arnica and natural menthol oil with cocoa butter and the scents of eucalyptus & lavender

**Examples of erroneous tweets (paraphrased slightly for anonymity). CBC: cannabichromene; CBD: cannabidiol; FDA: Food and Drug Administration; THC: tetrahydrocannabinol.**

If you live where medical marijuana is legal, get paid $3k a month to critique weed, CBD, edibles and more URL

The FDA is worried about CBD. Should you be concerned? URL

This room is half the size of my cbd apartment...

Flinders Street in Melbourne’s CBD has been re-opened following an earlier protest....Thanks for your patience during this disruption. #victraffic

Analysis of the manually annotated tweets indicated that the classes of personal and commercial CBD-related data sets were imbalanced; the nonpersonal CBD-related tweets occurred 7.7 times more than the personal CBD-related tweets, and the noncommercial CBD-related tweets occurred 10.2 times more than the commercial CBD-related tweets. To achieve a balance of the classes in the training set, we downsampled both of the positive classes in the training set by taking a random sample equivalent in size to the negative class. Table 1 and Table 2 show the class frequencies for the personal and commercial CBD-related tweet classes, respectively, prior to and following downsampling.

**Table 1:** Training set for personal cannabidiol (CBD) class counts.

Class	Predownsampling, n	Postdownsampling, n
Personal CBD	631	631
Nonpersonal CBD	4865	631
Total	5496	1262

**Table 2:** Training set for commercial cannabidiol (CBD) class counts.

Class	Predownsampling, n	Postdownsampling, n
Commercial CBD	489	480
Noncommercial CBD	45,007	489
Total	5496	978

### Classification Training

Before training the binary classifiers to sort the full data set into personal and commercial CBD-related tweets, we preprocessed the text of the tweets by normalizing all URLs to one consistent string, removing special characters and English part of speech, converting all of the text to lowercase, and lemmatization. This preprocessing was performed to reduce the noise in the data, which would potentially impact the performance of our tweet classifiers. The binary classifiers were then trained on 80% (4396 of 5496) of the annotated sample and validated on the remaining 20% (1099 of 5496) of the annotated sample. We then created a matrix of the term frequency-inverse document frequency (TF-IDF) features based on the words within tweets using a range of n-grams from 1 to 3, as well as a matrix of the TF-IDF features based on the characters within tweets using a range of n-grams from 3 to 6. The resulting matrices were stacked horizontally, which served as the input to our model for training the classifiers.

To train the two binary classifiers, we performed a 5-fold cross-validation grid search using a logistic regression model to find the optimal combination of parameters. The range of parameters is shown in Table 3. After training the binary classifiers, we applied each model to the larger CBD corpora of tweets. To compensate for the small validation set due to the balancing, we performed an additional postclassification test on a random set of 500 unbalanced tweets from our collection to confirm that our models would perform well on real-world unbalanced data. This sample was annotated with the same approach as used for the training set, with the predicted result hidden. We will discuss classification in further detail within the Results section.

**Table 3:** Parameters used in text classification tuning with the logistic regression model.

Parameter	Range
Penalty	{none,.l1.l2}
Regularization parameter	x_k_=10^a+(b-a)(k-1)/(n-1)^, k=1,…n; a=0; b=5; n=20
Solver	{newton-cg, lbfgs, liblinear, sag, saga}

### Term Analysis

To track the medical terminology referenced in the sorted commercial and personal CBD tweets, we computed the term frequencies of the top 1000 words in both corpora of tweets. We then confirmed whether these terms were related to relevant medical conditions, medical symptoms, body parts, and/or other medications/substances by referencing standard English, medical (Systemized Nomenclature of Medicine-Clinical Terms [SNOMED CT]), and slang dictionaries. We categorized the terms into three groups: health/medical, cannabis-related terms, and other substances. Within the health/medical group, we included terms related to diseases, aliments, symptoms, and body parts. We applied the same logic to the terms that appeared to be hashtags by examining the individual words that formed the hashtag for relevancy. We grouped cannabis-related terms together and separated them from the other substances group since there seemed to be an overlap of CBD- and THC-related tweets, both of which referenced the broader cannabis plant; we included cannabis-related slang terms as well as foods that are commonly associated with cannabis infusion (eg, gummies, honey). The other substances group included terms that refer to any other drug or medication. There were a few instances of words being included in multiple groups. For instance, “high” is a side effect of cannabis but is a term commonly used in both cannabis and CBD tweets. Additionally, we considered terms that may represent side effects caused by taking a substance, especially terms commonly associated with cannabis. Finally, we compared the overall frequency of the top occurring terms relative to their frequency in either the personal or commercial class of tweets, and produced a visualization of relevant term frequencies. We used the Scattertext Python package to generate a graphical representation of the frequencies within the personal and commercial CBD classes for each of the three term groups [30].

### Sentiment Analysis

We used the VADER model to compute the sentiment of the personal and commercial CBD tweets that reference specific medical conditions. Since this sentiment model incorporates punctuation and text capitalization into computing sentiment, we used the raw tweet text as an input for the model. The VADER model produced a normalized score between –1 and +1 for each tweet based on the summation of the valence scores of each word in the tweet. We converted each tweet’s score into a 3-level categorical variable based on the threshold recommended by Hutto and Gilbert [20]: (1) positive sentiment, compound score≥0.05; (2) neutral sentiment, compound score>–0.05 and compound score<0.05; and (3) negative sentiment, compound score ≤–0.05.

We then analyzed the distribution of the compound scores and sentiment class (positive, negative, neutral) in tweets containing terms related to specifically defined conditions and symptoms. Since the VADER model is partially based on a dictionary score and since many terms related to illness may influence the overall sentiment of a tweet (eg, pain, stress, cancer), as part of our analysis, we computed the VADER sentiment scores both with and without medical terms of interest, and compared the mean VADER scores using a *t* test to determine whether any individual term of interest biased the overall sentiment assigned to a tweet. The purpose of the *t* tests was to determine if there was a statistically significant difference in the sentiment conveyed in commercial CBD tweets containing certain terms versus the sentiment conveyed in personal CBD tweets referencing the same terms. For instance, the VADER sentiment score of “CBD really helps my pain” was –0.171 versus a VADER sentiment score of 0.4391 for “CBD really helps my,” where the word “pain” holds such a negative VADER sentiment score on its own that it is influencing the overall sentiment score of the tweet.

### Ethics

This study leverages publicly available data and is registered as approved by the University of Louisville Institutional Review Board (approved protocol 20.1122).

## Results

### Classification

We trained the binary classification algorithms for the personal and commercial CBD tweets independently. In both the optimal personal CBD classifier (logistic regression: C=3.36, penalty=none, solver=“newton-cg”) and commercial CBD classifier (logistic regression=428.13, penalty=“l1”, solver=“saga”), we observed a decrease in classification performance between the smaller validation set derived from balanced data from the unbalanced sample. Despite this decrease in classification performance on the unbalanced data, both the personal and commercial CBD classification models were able to achieve area under receiver operating characteristic curve scores above 0.80. Table 4 and Table 5 show the performance of the personal and commercial CBD binary classifiers, respectively. When the personal CBD binary classifier was applied to the collection of tweets, it classified 167,755 tweets as personal CBD-related tweets. When the commercial CBD binary classifier was applied to the collection of tweets, it classified 143,322 tweets as commercial CBD-related tweets.

**Table 4:** Personal cannabidiol (CBD) logistic regression classifier performance metrics.

Classification		Precision	Recall	F1	Support	Accuracy		AUC^a^	
**Balanced sample**							0.85		0.86
	Nonpersonal CBD	0.93	0.79	0.85	138				
	Personal CBD	0.79	0.93	0.85	115				
**Unbalanced sample**							0.89		0.87
	Nonpersonal CBD	0.94	0.91	0.93	367				
	Personal CBD	0.78	0.83	0.81	133				

^a^AUC: area under the receiver operating characteristic curve.

**Table 5:** Commercial cannabidiol (CBD) logistic regression classifier performance metrics.

Classifier			Precision		Recall		F1		Support		Accuracy			AUC^a^	
**Balanced sample**												0.89			0.89
	Noncommercial CBD	0.92		0.85		0.89		95							
	Commercial CBD	0.87		0.93		0.90		101							
**Unbalanced sample**												0.87			0.82
	Noncommercial CBD	0.90		0.93		0.91		367							
	Commercial CBD	0.79		0.70		0.74		133							

^a^AUC: area under the receiver operating characteristic curve.

### Term Analysis

We generated unigram frequencies for both the personal and commercial corpora of tweets. We looked at the top 1000 occurring terms (excluding common English stop words) and manually checked if the terms were relevant to health; wellness; diseases; side effects; conditions; body parts; and/or references to other substances against standard English, medical, and slang dictionaries.

**Figure 2:**
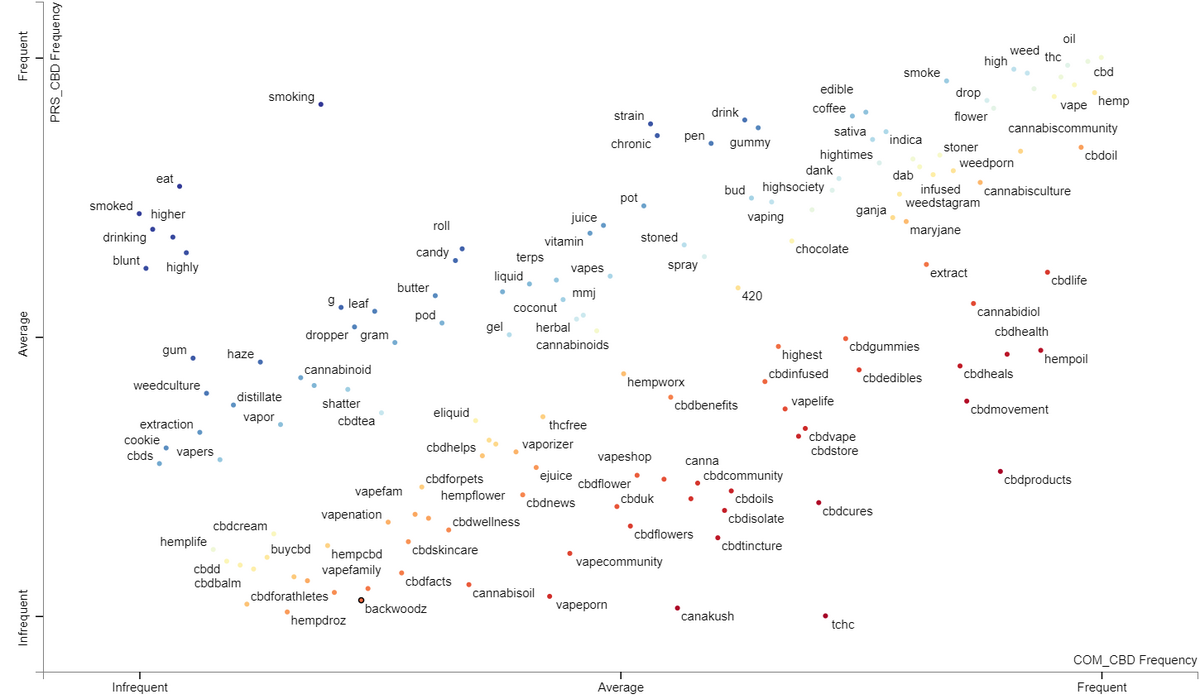
Cannabis-related term frequency per class.

**Figure 3:**
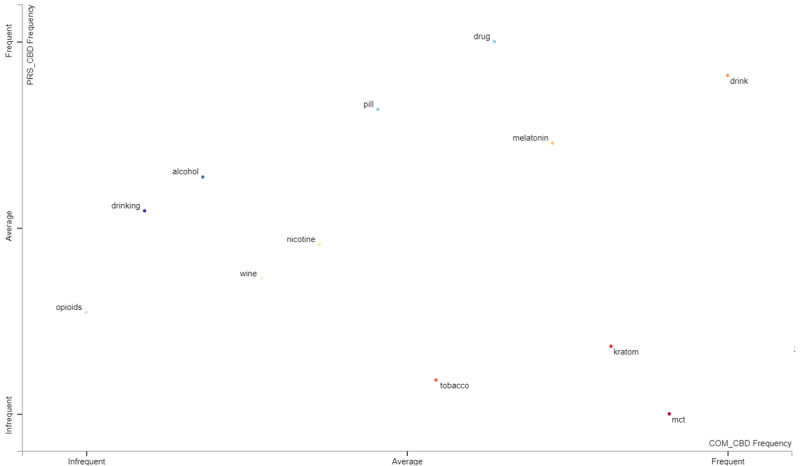
Other substances term frequency per class.

**Figure 4:**
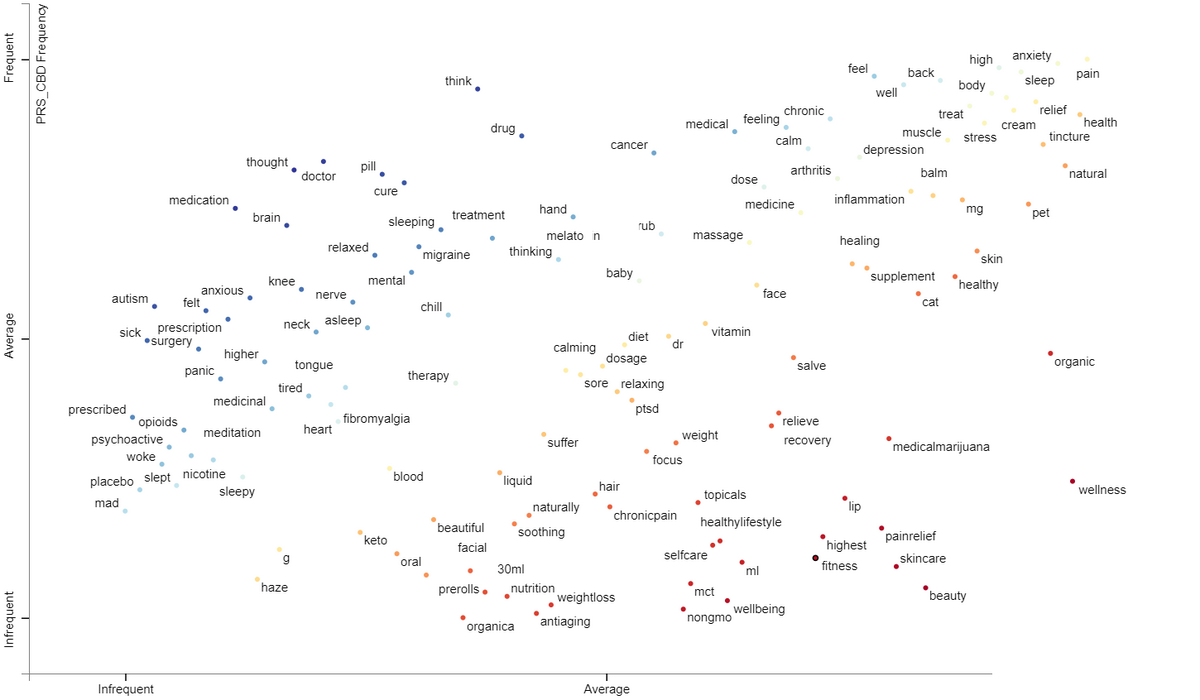
Medical/health/wellness-related term frequency per class.

In other tweets making cannabis references (Figure 2), it seemed that THC-related terms were mentioned in both the personal and commercial corpora of tweets, with hashtags containing these references more frequently found in the commercial CBD tweets. The terms drink, melatonin, and pills were mentioned in the other substances group (Figure 3) in both the personal and commercial CBD tweets. Kratom and medium-chain triglyceride (MCT) were mentioned more frequently within the commercial CBD tweets and less frequently in the personal CBD tweets. References to alcohol occurred slightly more than average within the personal CBD tweets and below average in the commercial CBD tweets. Opioids were mentioned but only infrequently in both the personal and commercial CBD tweet classes. In the health and wellness group (Figure 4), pain, sleep, and anxiety occurred frequently in both the personal and commercial CBD classes. Terms related to fitness and nutrition were found more frequently in the commercial CBD tweets. Tweets referencing posttraumatic stress disorder (PTSD) occurred at the same average within both classes. Finally, CBD tweets referencing autism occurred more frequently than average in personal tweets, but infrequently in commercial tweets. This is despite the US FDA sending out warning letters to CBD sellers for disseminating misinformation by promoting CBD as a treatment for a variety of medical conditions, including autism [29].

### Sentiment Analysis

We computed sentiment scores for both the commercial and personal CBD tweets that referenced any of the following 17 terms: *anxiety, anxious, autism, calm, calming, cancer, depression, energy, fitness, pain, pains, PTSD, skin, sleep, stress, weight loss,* and *wellness*. We also computed the term-level sentiment for each of these individual terms. Table 6 contains a list of the terms related to nonneutral sentiment and where the VADER score of the individual term might impact the sentiment score of the entire tweet. We calculated the sentiment for each personal and commercial CBD tweet referencing any of the 17 terms of interest, both in the original tweet text and with the term of interest removed. Using a *t* test to gauge any statistically significant difference in the mean sentient scores allowed us to assess how sentiments about the condition itself might affect the sentiment score.

**Table 6:** Medical-related terms with nonneutral sentiment.

Term	VADER^a^ compound score
anxiety	–0.1779
anxious	0.25
calm	0.3182
calming	0.4019
cancer	–0.6597
depression	–0.5719
energy	0.2732
pain	–0.5106
pains	–0.4215
stress	–0.4215

^a^VADER: Valence Aware Dictionary for Sentiment Reasoning.

**Figure 5:**
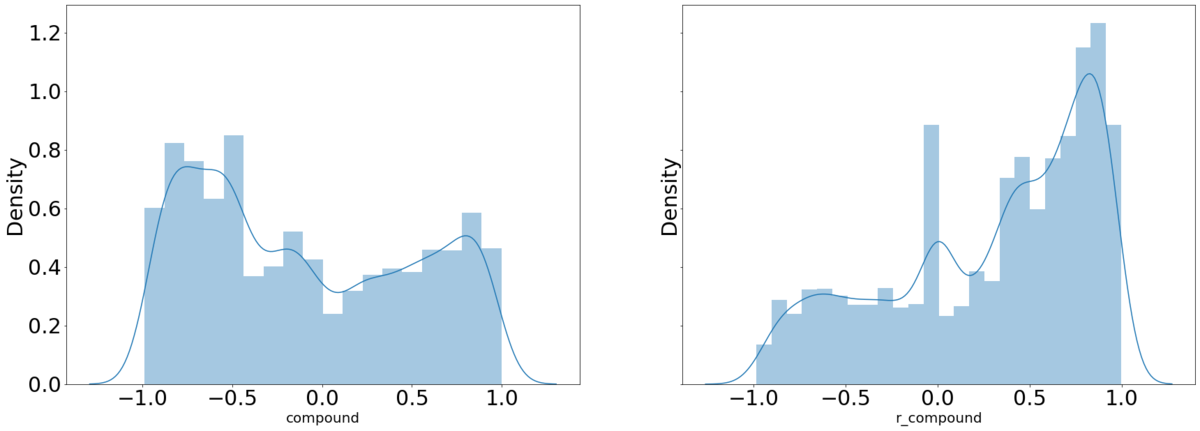
Distribution of sentiment scores of personal tweets referencing the term “pain.”.

**Figure 6:**
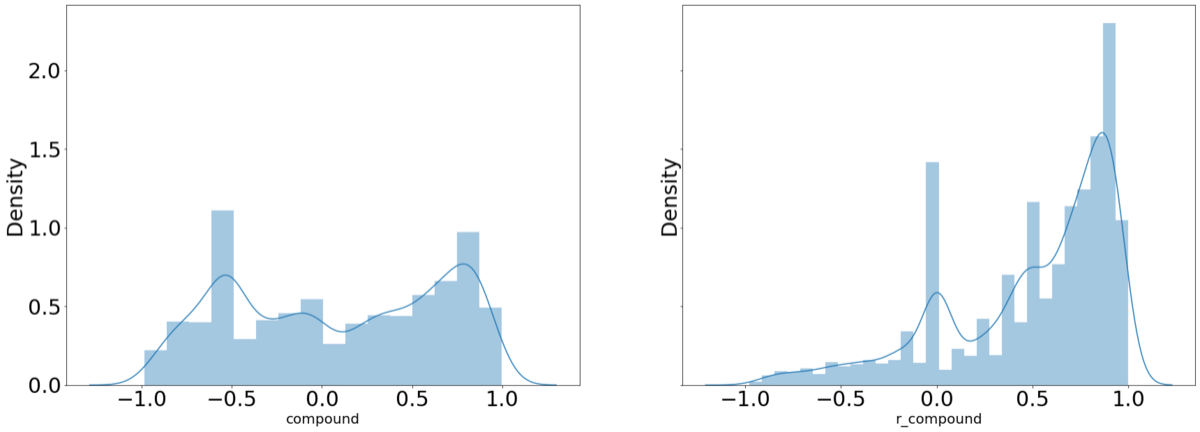
Distribution of sentiment scores of commercial tweets referencing the term “pain.”.

Tables 7-9 demonstrate a significant difference in the mean sentiment score in the personal CBD tweets with the term of interest versus that obtained without the term of interest for 11 of the 17 terms examined. There was a significant difference in the mean sentiment score in the commercial CBD tweets with the term of interest versus that obtained without the term of interest for 12 of the 17 terms examined. There was also a significant difference in the mean sentiment score in the commercial CBD tweets compared to that of the commercial CBD tweets with the term of interest for 11 of the 17 terms examined, both with and without the term of interest included. Table 8 indicates that although the sentiment was overall positive, in the instances where there was a significant difference in the sentiment scores between the personal and commercial CBD tweets, the mean sentiment score of the commercial CBD scores was higher than that of the personal CBD scores. Figure 5 and Figure 6 provide examples of how the distribution of the sentiment score changed when the term of interest (“pain”) was removed from the tweet.

**Table 7:** Personal and commercial cannabidiol (CBD) sentiment categorical counts.

Term	Personal tweets									Commercial tweets								
	n	With term				Without term				n		With term				Without term		
		pos^a^	neu^b^	neg^c^	pos		neu	neg			pos		neu	neg	pos		neu	neg
anxiety	5353	2818	126	2409	3125		519	1718	2924		1564		44	1316	1726		352	846
anxious	515	266	11	238	307		47	161	114		53		4	57	84		5	25
autism	395	180	47	168	180		47	168	27		17		2	8	17		2	8
calm	1224	1007	17	200	761		145	318	725		659		9	57	535		80	110
calming	445	399	4	42	324		33	88	389		369		2	18	308		47	34
cancer	986	230	19	737	530		111	345	246		76		0	170	122		44	80
depression	568	164	17	387	307		31	230	326		69		8	249	178		19	129
energy	507	416	9	82	334		57	116	444		421		7	16	357		52	35
fitness	57	48	2	7	37		4	16	128		125		0	3	100		15	13
pain	7432	2948	188	4296	4985		558	1889	6287		3262		113	2912	4956		591	740
pains	394	157	11	226	225		19	150	311		168		9	134	219		11	81
ptsd^d^	217	111	14	92	111		14	92	55		33		9	13	33		9	13
skin	618	461	55	102	464		54	100	2516		2211		150	155	2229		136	151
sleep	3761	2518	356	887	2517		356	888	2980		2129		322	529	2131		321	528
stress	1012	560	18	434	713		45	254	1407		883		28	496	1100		36	271
weight loss	8	5	2	1	5		2	1	24		18		3	3	18		3	3
wellness	144	129	2	13	98		18	28	4216		4020		38	158	3106		814	296

^a^pos: positive sentiment.

^b^neu: neutral sentiment.

^c^neg: negative sentiment.

^d^ptsd: posttraumatic stress disorder.

**Table 8:** Personal and commercial cannabidiol (CBD) sentiment score descriptive statistics (with and without the term).

Term	Personal tweets				Commercial tweets		
	n	With term, mean (SD)	Without term, mean (SD)	n		With term, mean (SD)	Without term, mean (SD)
anxiety	5353	0.074 (0.573)	0.186 (0.557)	2924		0.118 (0.568)	0.241 (0.538)
anxious	515	0.048 (0.591)	0.203 (0.566)	114		0.08 0 (0.566)	0.254 (0.529)
autism	395	–0.001 (0.546)	–0.001 (0.546)	27		0.188 (0.557)	0.188 (0.557)
calm	1224	0.448 (0.484)	0.258 (0.540)	725		0.616 (0.374)	0.452 (0.467)
calming	445	0.608 (0.408)	0.410 (0.508)	389		0.695 (0.334)	0.513 (0.444)
cancer	986	–0.369 (0.571)	0.158 (0.559)	246		–0.303 (0.638)	0.167 (0.564)
depression	568	–0.275 (0.573)	0.122 (0.571)	326		–0.353 (0.506)	0.111 (0.525)
energy	507	0.469 (0.492)	0.324 (0.541)	444		0.681 (0.331)	0.547 (0.421)
fitness	57	0.429 (0.463)	0.263 (0.514)	128		0.633 (0.283)	0.464 (0.400)
pain	7432	–0.099 (0.605)	0.293 (0.547)	6287		0.088 (0.580)	0.490 (0.440)
pains	394	–0.098 (0.610)	0.169 (0.577)	311		0.087 (0.615)	0.342 (0.553)
ptsd^a^	217	0.037 (0.626)	0.037 (0.627)	55		0.200 (0.563)	0.200 (0.563)
skin	618	0.420 (0.501)	0.427 (0.501)	2516		0.550 (0.371)	0.568 (0.371)
sleep	3761	0.305 (0.522)	0.305 (0.523)	2980		0.392 (0.493)	0.394 (0.493)
stress	1012	0.116 (0.632)	0.360 (0.567)	1407		0.234 (0.596)	0.481 (0.396)
weight loss	8	0.289 (0.344)	0.289 (0.344)	24		0.436 (0.549)	0.436 (0.549)
wellness	144	0.606 (0.431)	0.384 (0.524)	4216		0.720 (0.279)	0.505 (0.426)

^a^ptsd: posttraumatic stress disorder.

**Table 9:** Personal and commercial cannabidiol (CBD) sentiment score t test results (with and without term).

Term	Personal with vs without term			Commercial with vs without term			Commercial vs personal with term			Commercial vs personal without term		
	*t*	*df*	*P* value	*t*	*df*	*P* value	*t*	*df*	*P* value	*t*	*df*	*P* value
anxiety	–10.31	10,704	<.001	–8.51	5846	<.001	–3.33	8275	.001	–4.28	8275	<.001
anxious	–4.29	1028	<.001	–2.39	226	.02	0.53	627	.59	–0.88	627	.38
autism	0.00	788	>.99	0.00	52	>.99	–1.74	420	.08	–1.74	420	.08
calm	9.15	2446	<.001	7.40	1448	<.001	–8.06	1947	<.001	–8.04	1947	<.001
calming	6.40	888	<.001	6.49	776	<.001	–3.37	832	.001	–3.09	832	.002
cancer	–20.71	1970	<.001	–8.65	490	<.001	–1.59	1230	.11	–0.22	1230	.83
depression	–11.67	1134	<.001	–11.49	650	<.001	2.06	892	.40	0.29	892	.77
energy	4.45	1012	<.001	5.25	886	<.001	–7.68	949	<.001	–7.02	949	<.001
fitness	1.81	112	.07	3.92	254	<.001	–3.69	183	<.001	–2.88	183	.004
pain	–41.39	14,862	<.001	–43.82	12,572	<.001	–18.37	13,717	<.001	–23.02	13,717	<.001
pains	–6.32	786	<.001	–5.43	620	<.001	–3.98	703	<.001	–4.01	703	<.001
ptsd^a^	–0.01	432	.99	0.00	108	>.99	–1.77	270	.08	–1.76	270	.08
skin	–0.24	1234	.81	–1.76	5030	.08	–7.25	3132	<.001	–7.89	3132	<.001
sleep	0.01	7520	.99	–0.12	5958	.90	–6.97	6739	<.001	–7.10	6739	<.001
stress	–9.12	2022	<.001	–11.98	2812	<.001	–4.65	2417	<.001	–5.60	2417	<.001
weight loss	0.00	14	>.99	0.00	46	>.99	–0.71	30	.48	–0.71	30	.48
wellness	3.94	286	<.001	27.38	8430	<.001	–4.72	4358	<.001	–3.35	4358	.001

^a^ptsd: posttraumatic stress disorder.

**Figure 7:**
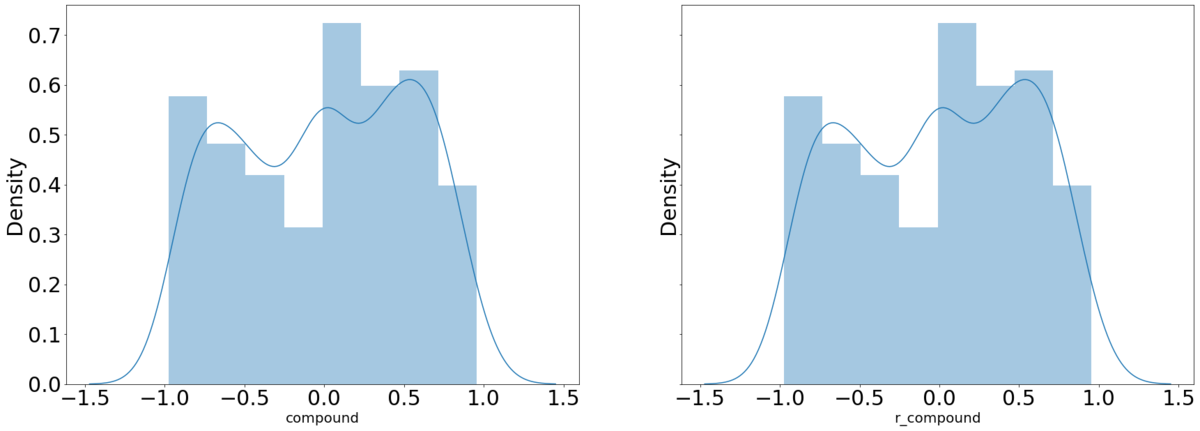
Sentiment distribution of personal cannabidiol-related tweets referencing autism.

There were instances of CBD tweets that offered a mix of both positive and negative sentiments within the personal tweets, such as in tweets referencing CBD’s relationship with autism. Figure 7 shows a more negative sentiment in the personal CBD tweets referencing autism. However, the sentiment of the personal tweets did not change when the term “autism” was removed. Despite being negative, the mean sentiment score of these tweets was –0.042, which is considered neutral by the authors of the VADER model. We observed a large amount of personal CBD tweets referencing the term “autism,” with 42.5% (168/395) of personal CBD tweets referencing autism classified as negative versus 45.6% (180/395) being classified as positive and 11.9% (47/395) classified as neutral.

Textbox 3 shows tweets that our classifiers identified to be CBD-related that contained the word “autism.” These personal CBD tweets sometimes favored and sometimes disfavored CBD as a treatment for autism. In the commercial CBD tweets referencing autism, we observed both implicit and explicit claims regarding CBD’s ability to treat autism.

Our framework thus works well in contexts where the efficacy claims of medications and supplements are both validated and refuted.

**Examples of personal and commercial tweets referencing cannabidiol (CBD) and autism (paraphrased slightly for anonymity).**

**Personal autism CBD tweets**

@user @user He’s...on a thc/cbd tincture. It’s helped his autism beautifully

@user I use CBD for my C-PTSD [posttraumatic stress disorder] and the overstimulation that comes from Autism...it’s kinda hard to function...It works better than any antipsychotic I’ve ever been on.

I see a lot of ppl on my timeline...claiming CBD can “cure” autism is bad and so is anyone knowingly peddling the idea what is wrong with you people URL

@user @user @user if you work somewhere that lies about curing autism with cbd oil you should feel bad

**Commercial autism CBD tweets**

10 Best CBD Oils For Autism - URL 10-bestcbd-oils-for-autism-40/

CBD INFUSED … BOTTLES @ FRUIT PASTELS, THESE ARE IDEAL FOR KIDS WITH ADHD, AUTISM ETC #THECBDCHEMIST #GOODNIGHTSLEE

COMPANY_NAME® Announces Autism Hope Alliance Sponsorship URL #cannabis #hemp #cbd #vape #cbdoil #natural #anxiety #pain #stress #health #pharma #wellness #beauty #domains URL

People use CBD to treat everything from epilepsy and autism to chronic pain and anxiety. URL

## Discussion

### Principal Findings

Text classification of tweets provides a means to segment tweets into defined groups at a large scale. We have demonstrated that we can do this with tweets related to CBD by using text classification to identify tweets that reflect personal usage of CBD and tweets that reflect the sales and/or commercialization of CBD. This classification of public social media data is useful because CBD has not been subjected to the same tests and clinical trials as modern medications, yet is currently being used to treat a variety of conditions without proof of safety or efficacy. Our analysis provided a methodology to identify the terms of interest that are frequently referenced in the commercial and personal corpora of CBD tweets, as well as a comparison of these term frequencies in relation to the document class (commercial or personal CBD). This allowed us to identify the medical conditions commonly referenced in both document classes at high frequencies, as well as terms that occur more frequently in one document class over the other. We also used the VADER model to analyze the sentiment of personal CBD tweets referencing certain medical conditions and symptoms. Despite the US FDA’s warnings regarding the marketing and promotion of CBD as treatment of autism and Alzheimer disease, while certainly not the most frequent conditions referenced, we did observe multiple instances of these tactics.

These methods and results speak to the recent efforts of researchers to use social networks to analyze the concept of misinformation in ways that are directly related to the potential problem of CBD misinformation. Ferrand et al [25] analyzed responses to queries from common digital assistants such as Siri, Alexa, and Google for misinformation regarding vaccines. Chen et al [26] collected social network posts from Weibo related to cancer and observed that 30% of posts contained misinformation. Ahmed et al [27] collected tweets referencing COVID-19 and 5G, and performed graph analysis to identify and analyze how misinformation was being disseminated online. Allem et al [3] observed unsubstantiated health claims related to cannabis on Twitter. More recently, Rovetta and Bhagavathula [28] also observed an abundance of COVID-19 misinformation in their analysis of tweets.

To address potential social media misinformation in the area of CBD, it is important to develop methods for gathering and classifying text corpora. Previous studies using the internet and social media have described the personal and commercial discourses about CBD. Narayanan et al [15] made use of internet-based data sources to examine CBD trends by examining Google searches, demonstrating that interest in CBD oil increased significantly from 2014 to 2018. Tran and Kavuluru [31] used CBD-related posts from Reddit and comments submitted to the FDA regarding these posts to examine the conditions that are commonly being treated by CBD. The researchers in this study examined both corpora of texts for medical conditions and methods of use in posts and comments using the term “CBD,” along with any indication of therapy implied in the two corpora.

There have also been nonmachine learning approaches to researching marijuana sentiment on Twitter, such as the work done by Nguyen et al [32]. Their study collected marijuana-related tweets, disregarded the tweets that were authored by less influential posters, manually annotated the marijuana tweets on a Likert scale via crowdsourcing, and segmented them by demographics applied to the data set through a proprietary service. The researchers observed more promarijuana attitudes among African Americans and youth/younger adults. In another example of a research approach that did not rely primarily on machine learning, Krauss et al [33] based their marijuana sentiment analysis on crowdsourced tweets. The researchers aimed to examine the preferences between marijuana and alcohol on Twitter. They collected tweets containing alcohol and marijuana references, and then annotated the tweets via crowdsourcing. The results showed that 54% of the tweets normalized marijuana and alcohol, 24% showed a preference for marijuana over alcohol, 2% showed a preference for alcohol over marijuana, 7% showed negative sentiment toward both alcohol and marijuana, and 13% demonstrated no sentiment toward either substance.

Our proposed framework extends the existing CBD research by further examining the perceptions of CBD in online discussions through a comparison of the terms and sentiment of tweets that reflect the personal use of CBD and tweets that reflect the sales and/or promotion of CBD. No other studies have attempted this type of comparative work, and this approach helped to examine which terms are being used proportionally or disproportionally, and to compare the sentiment of personal and commercial CBD tweets. Our methods can be applied to other types of research aimed at analyzing trends in the consumption and advertising of unregulated substances.

### Conclusion and Future Work

The strengths of our framework are the ability to identify personal and commercial CBD tweets, associated conditions, and sentiment. However, some limitations should be noted. First, we limited our search to tweets referencing “CBD” and “cannabidiol.” Our preliminary research did not indicate that the topic of CBD is as subjected to slang terms as other forms of cannabis (eg, THC) and did not indicate that it was necessary to include additional related terms in the search [34]. Additionally, we limited our collection to tweets as Twitter is one of the world’s largest social networks that provides the ability to collect a large volume of data quickly. Second, our data were collected over an approximate 3-month period. Although the data collection period was relatively small, we were able to identify trends in personal and commercial CBD tweets that will be useful for future studies. Another limitation is the use of the dictionaries (standard, slang, and SNOMED-CT) for finding associated medical conditions. This step was based on checking high-frequency terms against dictionaries to determine medical relevancy. Future research could use deep neural network models to extract medical-related named entities from the tweets to automate and to possibly obtain context in which the medically related term is being used [35]. Finally, although we did not explicitly identify and remove social bots from our collection, as discussed by Himelein-Wachowiak et al [36], we did remove bots during the annotation process, as tweets that were possibly machine-generated were not considered personal or commercial CBD tweets.

We successfully used text classification to identify tweets making personal or commercial CBD references. When we applied two classifiers to the collection of tweets, we identified multiple medical conditions, body parts, symptoms, other substances, and cannabis references that were mentioned at high frequencies in both the personal and commercial CBD corpora, as well as conditions that were mentioned disproportionately in one corpora over the other. This suggests that CBD is being used and marketed for consistent types of ailments. Our sentiment analysis showed that the term of interest can indeed influence the sentiment score; when controlling for the term, 15 of 17 terms tested showed a positive sentiment within the personal CBD tweets and all 17 terms showed a positive sentiment within the commercial CBD tweets. This suggests that CBD, on the whole, is well-regarded in terms of its medical applications and that the commercial claims are not gross distortions of popular sentiment; however, we observed evidence where claims may have been exaggerated. We encourage future research to investigate the patterns in sentiment, usage, and sales of CBD as well as other forms of cannabis over time. Additionally, we recommend extending this proposed framework by further using text mining and machine learning methods to identify the dissemination of misinformation as it relates to CBD’s health and medical benefits.

## Abbreviations

CBC: cannabichromene
CBD: cannabidiol
FDA: Food and Drug Administration
MCT: medium-chain triglyceride
PTSD: posttraumatic stress disorder
SNOMED-CT: Systemized Nomenclature of Medicine-Clinical Terms
TF-IDF: term frequency-inverse document frequency
THC: tetrahydrocannabinol
VADER: Valence Aware Dictionary for Sentiment Reasoning
